# Translational value of IDH1 and DNA methylation biomarkers in diagnosing lung cancers: a novel diagnostic panel of stage and histology-specificity

**DOI:** 10.1186/s12967-019-2117-7

**Published:** 2019-12-30

**Authors:** Ruochuan Zang, Xinfeng Wang, Runsen Jin, Yuanyuan Lei, Jianbing Huang, Chengming Liu, Sufei Zheng, Fang Zhou, Qian Wu, Nan Sun, Shugeng Gao, Jie He

**Affiliations:** Department of Thoracic Surgery, National Cancer Center/National Clinical Research Center for Cancer/Cancer Hospital, Chinese Academy of Medical Sciences and Peking Union Medical College, Beijing, 100021 China

**Keywords:** Lung cancer, Diagnostic biomarker, IDH1, DNA methylation, Circulating tumor DNA

## Abstract

**Background:**

Lung cancer is the leading cause of cancer-related death worldwide, and the timely and serial assessment of low-dose computed tomography (LDCT) in high-risk populations remains a challenge. Furthermore, testing a single biomarker for the diagnosis of lung cancers is of relatively low effectiveness. Thus, a stronger diagnostic combination of blood biomarkers is needed to improve the diagnosis of non-small cell lung cancer (NSCLC).

**Methods:**

The blood levels of individual biomarkers [IDH1, DNA methylation of short stature homeobox 2 gene (SHOX2), and prostaglandin E receptor 4 gene (PTGER4)] were measured and statistically analyzed in samples from healthy controls and patients with lung cancer. In total, 221 candidates were enrolled and randomly assigned into two groups for the training and validation of a diagnostic panel. Additionally, a subgroup analysis was performed in the whole cohort.

**Results:**

A newly combined 3-marker diagnostic model for lung cancers was established and validated with area under the receiver operating characteristic (ROC) curve (AUC) values ranging from 0.835 to 0.905 in independent groups showing significantly stronger diagnostic value compared with a single tested biomarker. The sensitivity of the diagnostic model was as high as 86.1% and 80.0% in the training and validation sets, respectively. Although no apparent differences were found between the 3-marker and 2-marker models, the high clinical T-stage and histological type specificity of IDH1 and two other methylated DNA biomarkers were demonstrated in the subgroup analysis.

**Conclusions:**

The combination of single biomarkers with high stage-specificity and histological type specificity (SHOX2 and PTGER4 DNA methylation and IDH1) showed better diagnostic performance in the detection of lung cancers compared with single marker assessment. A greater clinical utility of the panel may be developed by adding demographic/epidemiologic characteristics.

## Background

Despite great progress in the surgical and medical management of malignant tumors, lung cancer still ranks first as the most commonly diagnosed malignant disease and is the leading cause of cancer death for both males and females worldwide [1]. Although the randomized National Lung Screening Trial has demonstrated the advantage of low-dose computed tomography (LDCT) compared to chest X-rays for screening lung cancers [2], annual screening was performed on only 4% of patients in high-risk populations [3]; moreover, its relatively lower specificity for indeterminate lung nodules, poor repeatability and related economic burden on the public health system increased the diagnostic cost for lung cancers. Owing to the untimely diagnosis and limited treatment options, the 5-year survival rate for advanced-stage lung cancers is only 5%, which is extremely low compared to localized and regional stage lung cancers (56% and 29%, respectively) [4]. Thus, developing a novel diagnostic panel is of priority to improve the treatment modality and survival rate of lung cancer patients.

As the discriminative diagnostic potential of biological fluid-based biomarkers for the detection of lung cancers has been shown [5–9], the improvement of cancer management and treatment have increasingly relied on a combination of biomarkers. Abnormal epigenetic events are suggested to occur in the early phase of carcinogenesis [10, 11], making DNA methylation biomarkers available for the early detection and monitoring of cancers [12, 13]. Based on previous studies, short stature homeobox 2 (SHOX2) gene DNA was confirmed to have higher methylation levels in patients with small cell lung cancer (SCLC) and squamous cell carcinoma (SCC) than in patients with adenocarcinoma (Ade) and in healthy controls [14–16], indicating its potential as a diagnostic biomarker for specific types of lung cancers. Additionally, other clinical applications such as the assessment of lung cancer stages by measuring the SHOX2 methylation levels of lymph nodes [17] and its efficiency for monitoring the response to chemotherapy [18] have been studied. The prostaglandin E2 receptor 4 gene (PTGER4) product, EP4, is important in tumor progression mediated by prostaglandin E2 [19–21], which has been confirmed to have an upregulated expression in cancer tissues compared with that in normal tissues [20, 22]. Although, the discriminative ability in detection of lung cancers of the SHOX2/PTGER4 DNA methylation marker panel has been confirmed. However, it was concluded in a population containing relative higher percentage of non-Ade lung cancers (Ade: non-Ade, 46:71) [23]. Thus, in our present study, the two methylation DNA biomarkers were enrolled as candidate markers for analysis in a population mainly containing lung Ades. In addition, isocitrate dehydrogenase 1 (IDH1) has been proven to have an important role in promoting tumor growth in NSCLC [24] and could be used as a blood biomarker for the detection of NSCLC, particularly lung Ade [25].

From the perspective of building a marker panel with high diagnostic efficiency and wide coverage of lung cancers, the plasma levels of protein and methylation levels of circulating DNA were measured and analyzed.

## Methods

### Blood samples and patient details

Between January 2017 and December 2018, 221 candidates from the Cancer Hospital of the Chinese Academy of Medical Sciences were enrolled in our study and randomly divided into the training and validation groups. Blood samples were collected from lung cancer patients who met the following criteria: (a) no history of other specific malignant diseases; and (b) no anticancer treatments before the blood sample collection process. Healthy individuals in the control group were selected from Physical Examination Centers during the same period; these individuals were confirmed to have no lung nodules by chest X-ray or thin-sliced computed tomography as well as no history of malignant tumors. This study was conducted following national institutional ethical policies and approved by the Chinese Academy of Medical Sciences Institutional Review Board.

### DNA preparation and bisulfite conversion from plasma specimens

To ensure objectivity, laboratory personnel were blinded to the identities of the samples. For each subject, 10 mL of blood was collected in 10 mL BD Vacutainer ethylenediaminetetraacetic acid tubes (BD Biosciences, San Jose, CA). Each tube was centrifuged for 12 min at 1350×*g* ± 150×*g* at room temperature. Plasma was transferred without disturbing the buffy coat to a clean 15 mL conical tube. The sample was centrifuged a second time for 12 min at 1350×*g* ± 150×*g*. Plasma was transferred without disturbing the pellet to a clean 15 mL conical tube and stored at − 20 °C. If not assayed immediately, plasma was stored at − 20 °C for up to 2 weeks. The DNA extraction from plasma samples, bisulfite conversion, and purification were performed using the Plasma Preparation Kit (Biochain (Beijing) Technology Co., Ltd., Beijing, China). DNA was eluted in 60 μL of elution buffer. If not used immediately, the eluted DNA was stored at − 20 °C for up to 3 days.

### Real-time PCR

The PCR method was performed as described in the kit for gene methylation (real-time PCR) (Biochain Technology Co., Ltd., Beijing, China). PCR amplification was performed in triplicate for each sample. SHOX2, PTGER4 and beta-actin (ACTB) control reactions were performed in the same reaction. Real-time PCR was performed on a 7500 Real-time PCR instrument (Applied Biosystems, CA) using the following cycling conditions: activation at 95 °C for 20 min, 45 cycles of 62 °C for 5 s, 55.5 °C for 35 s, 93 °C for 30 s and final cooling to 40 °C for 5 s. The heating rates were 2.4 °C/s, and the cooling rates were 2.4 °C/s. Data were acquired at the end of each 55.5 °C step. Analysis was performed using the ABI 7500 SDS software V2.0.5.

### ELISA detection of serum IDH1

For each individual, 4 mL of blood was collected in 4 mL BD Vacutainer ethylenediaminetetraacetic acid tubes (BD Biosciences, San Jose, CA) and then centrifuged at 3000×*g* for 5 min. The supernatant was divided into 500 μL aliquots, stored at − 80 °C and detected simultaneously. The detection of serum IDH1 levels was conducted with an ELISA kit (Biochain Technology Co., Ltd., Beijing, China) following the manufacturer’s instructions. Before the assay, the serum samples and test kits were equilibrated to room temperature. Then, 50 μL of each sample was added to the appropriate wells and covered with a film. The plate was incubated at 37 °C for 2 h. The liquid was discarded from each well, and the plate was washed 5 times. Then, 50 μL of testing solution A was added to each well and covered with a film. The plate was incubated at 25 °C for 1 h. After 5 washes with buffer, 50 μL of testing solution B was added to each well, and the plate was incubated at 25 °C for 1 h. Then, 50 μL of mixed substrates A and B were added to each well, and the plate was incubated at 25 °C for 15 min. To stop the reaction, 50 μL of stop solution was added. The optical density (O.D.) was detected at 450 nm on a Multiskan FC microplate photometer (ThermoFisher Scientific, Waltham, USA). The concentration of IDH1 was calculated with a quadratic polynomial fitting curve.

### Statistical analysis of the data

The Mann–Whitney U test or t test was used for the statistical analysis of biomarker levels between the normal controls and lung cancer patients. To build a predictive model for the discrimination of lung cancers, the forward logistic regression method was used to screen variables. The area under the receiver operating characteristic (ROC) curve (AUC) with a 95% confidence interval (*CI*) was applied to compare the diagnostic performance of single markers and the model. In addition, Youden’s index of the training group was used to determine the cut-off value of the predictive model. The diagnostic performances of our model in different subgroups were analyzed by comparing the AUCs of the ROC curves. Other descriptive statistics, such as sensitivity, specificity, positive predictive value (PPV), negative predictive value (NPV) and standard deviation (SD), were calculated in this study.

Software including SPSS 24.0, GraphPad Prism 5.0, MedCalc (version 11.4.2.0) and Microsoft Excel were used for statistical analysis. *p* values were two tailed. Differences were considered statistically significant with a *p* value less than 0.05.

## Results

### Basic characteristics of the two randomly assigned groups

In our study, the whole enrolled population was randomly assigned into the training and validation groups. There were 170 candidates (55 healthy controls and 115 patients with lung cancers) in the training group, and the remaining 16 healthy controls and 35 lung cancer patients were randomly selected as the validation cohort. In both groups, the ratios of healthy controls to patients were near 1:2. And, in the training and validation groups, most patients (78.3% and 62.9%, respectively) had a diagnosis with lung Ade. Furthermore, the enrolled population was divided according to tumor size and pathological type, as shown in Table 1, to analyze the diagnostic performances of subgroups. Other basic information is shown in Table 1.

**Table 1:** Basic information

Variables	Training group	Validation group	Whole group
	(n = 170)	(n = 51)	(n = 221)
Sex			
Male	71	31	102
Female	99	20	119
Age			
≤ 60	125	39	164
> 60	45	12	57
Tumor size			
≤ 5 cm	94	29	123
> 5 cm	21	6	27
Pathology			
Ade	90	22	112
SCC	14	5	19
SCLC	9	6	15
Others	2	2	4
Healthy controls	55	16	71
Molecular subtypes			
EGFR MUTs (+)	46	13	59
KRAS MUTs (+)	2	3	5
No MUTs detected	31	3	34
Not available	36	16	52

Detected subtypes of EGFR MUTs (+) defined as commonly observed point mutations of exon 18 and/or deletions of exon 19 and/or exon 21 insertion mutations and/or point mutation in exon 21 (L858R); KRAS MUTs (+) defined as mutations in codons 12 and/or 13 of exon 2

*Ade* adenocarcinoma, *SCC* squamous cell cancer, *SCLC* small cell lung cancer, *EGFR* epidermal growth factor receptor, *MUTs* mutations

### Basic analysis of single biomarkers in the training and validation groups

**Fig. 1:**
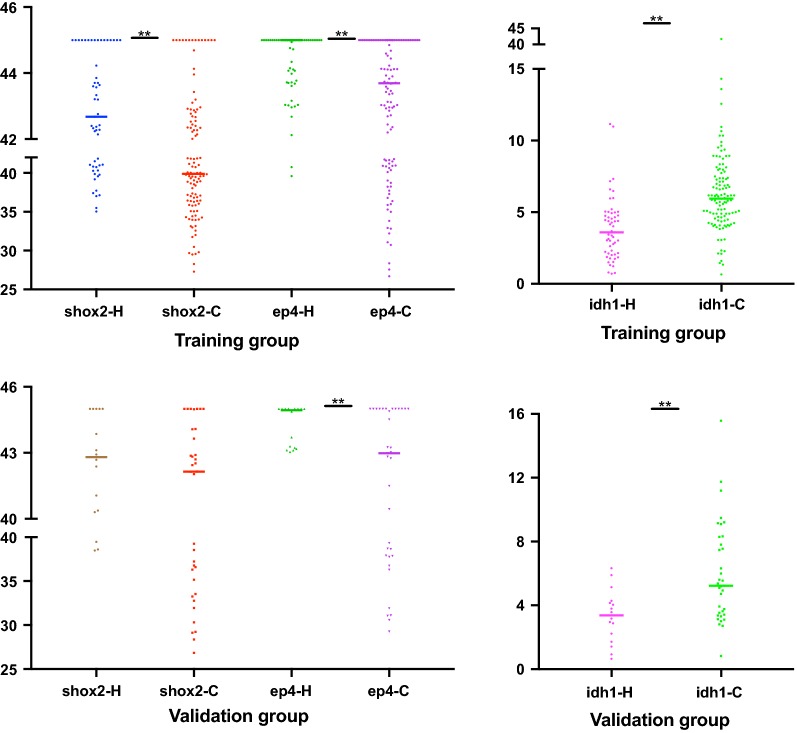
Comparisons of biomarkers between lung cancer patients and healthy controls in the training and validation cohorts. *Shox2* short stature homeobox 2 gene, *ep4* prostaglandin E2 receptor 4 gene (PTGER4), *idh1* isocitrate dehydrogenase 1, *y-axis* cycle threshold, *H* healthy controls, *C* lung cancer patients, **p value < 0.05

The levels of single biomarkers in lung cancer patients and healthy controls, as shown in Fig. 1, were separately assessed in the two cohorts. In the training group, the methylation levels of SHOX2 and PTGER4 in cancer patients were significantly higher than those in healthy controls (*p *< 0.05). In addition, the levels of IDH1 in lung cancer patients were significantly elevated (*p *< 0.05).

Similar conditions in the validation group, i.e., significantly increased methylation levels of PTGER4 and elevated levels of IDH1, were observed in lung cancer patients compared with healthy controls (*p *< 0.05). Although nonsignificant, the methylation levels of SHOX2 in patients with lung cancers were higher than those in healthy controls.

### Construction, analysis and validation of the three-biomarker diagnostic panel in two independent groups

By testing the levels of the three biomarkers together, a predictive model was built based on the results of the forward logistic regression analysis (Additional file 1: Table S1). As a result, all three measured biomarkers were enrolled, contributing to the final panel = 16.821 + 0.435 * IDH1 − 0.147 * SHOX2 − 0.28 * PTGER4.

**Fig. 2:**
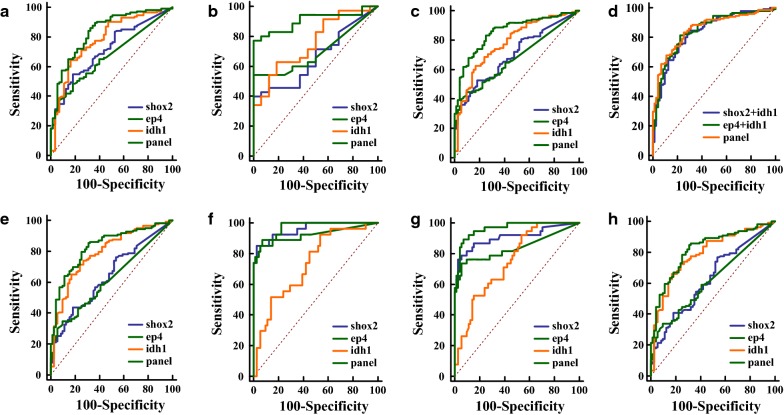
Receiver operating characteristic (ROC) curves for each single tested biomarker and diagnostic model in different cohorts. *Shox2* short stature homeobox 2 gene, *ep4* prostaglandin E2 receptor 4 gene (PTGER4), *idh1* isocitrate dehydrogenase 1. **a**–**c** Comparison of the diagnostic ability of the primary 3-marker model (IDH1 + SHOX2 + PTGER4) and each biomarker in the training cohort, the validation cohort, and the whole cohort, respectively. **d** Analysis of the diagnostic ability of the primary 3-marker model and the 2-marker models in the whole cohort. **e**–**h** Subgroup analysis of the model in the whole cohort. **e**, **f** Diagnostic performance in the group of early stage lung cancers and advanced-stage lung cancers, respectively. **g**, **h** ROCs of the primary model in the non-Ade lung cancer group (**g**) and Ade lung cancer group (**h**)

The diagnostic abilities of the individual biomarkers and the panel were analyzed in three groups separately (Table 2). Among the three enrolled tested biomarkers, IDH1 had the highest AUCs of 0.781 (95% *CI* 0.711–0.841), 0.755 (95% *CI* 0.615–0.865), and 0.78 (95% *CI* 0.719–0.833) in the training, validation and whole group, respectively (Fig. 2a–c). However, in the training cohort, the diagnostic panel was confirmed with the highest AUC of 0.835 (95% *CI* 0.770–0.887), which was significantly better than any single marker (*p *< 0.05). In addition, the panel exhibited the highest AUCs of 0.905 (95% *CI* 0.790–0.969) and 0.856 (95% *CI* 0.803–0.899) in the validation group and the whole group, respectively, demonstrating a significantly stronger diagnostic value compared with any single candidate biomarker (*p *< 0.05, Table 2).

**Table 2:** Comparisons of diagnostic models and individual markers in different groups

Group	Variables	AUC	S.E.	95% CI	*p* value
Training group (n = 170)	shox2	0.714	0.0398	0.640–0.781	0.0011
	ep4	0.67	0.0391	0.594–0.740	< 0.0001
	idh1	0.781	0.0385	0.711–0.841	0.0207
	panel	0.835	0.033	0.770–0.887	–
Validation group (n = 51)	shox2	0.659	0.0767	0.513–0.786	0.0009
	ep4	0.699	0.0684	0.555–0.820	0.0017
	idh1	0.755	0.0703	0.615–0.865	0.015
	panel	0.905	0.0419	0.790–0.969	–
Whole group (n = 221)	shox2	0.7	0.0351	0.634–0.759	< 0.0001
	ep4	0.674	0.0338	0.608–0.735	< 0.0001
	idh1	0.78	0.033	0.719–0.833	0.0018
	panel	0.856	0.0258	0.803–0.899	–

*shox2* short stature homeobox 2 gene, *ep4* prostaglandin E2 receptor 4 gene (PTGER4), *idh1* isocitrate dehydrogenase 1, *AUC* area under the curve, *S.E.* standard error, *95% CI* 95% confidence interval

**Fig. 3:**
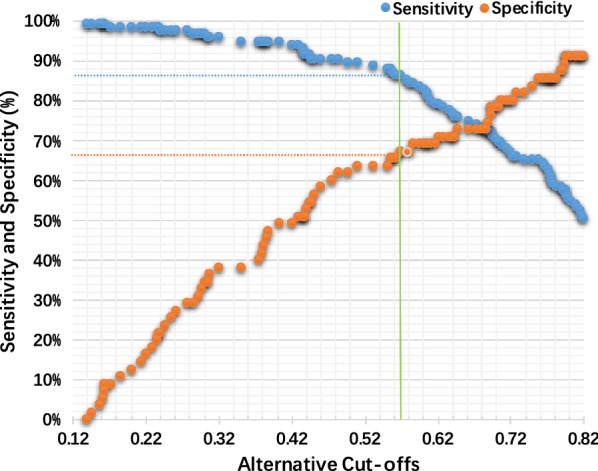
Resulting sensitivity and specificity of the primary 3-marker diagnostic model in the training group when using the alternative cut-offs. A cut-off of 0.596 was selected with a specificity of 67.3% and a sensitivity of 86.1%

In this study, a Youden’s index of 0.534 was chosen to select the cut-off value, 0.569 (Fig. 3). Based on the cut-off value of the diagnostic model, the sensitivity of the training group and validation group were calculated as 86.1% and 80.0%, respectively. The specificity of the model in the validation group was as high as 87.5%; however, it was relatively lower in the training group, 67.3%. In addition, the high diagnostic accuracy, 80.0% and 82.4% in the training and validation groups, respectively, demonstrated its clinical advantage in discriminating lung cancers and healthy controls over any single biomarker. Other statistical information is specifically listed in Table 3.

**Table 3:** Basic assessment of the model in the training and validation groups

Group	Cancer patients	Healthy controls	Se (%)	Sp (%)	NPV (%)	PPV (%)	Accuracy (%)
Training group	115	55	86.1	67.3	70.0	84.6	80.0
(n = 170)							
Validation group	35	16	80.0	87.5	66.7	93.3	82.4
(n = 51)							

*Se* sensitivity, *Sp* specificity, *NPV* negative predictive value, *PPV* positive predictive value

### Analysis of the diagnostic model with different histological subtypes and T-stages in the whole cohort

To investigate the diagnostic ability with respect to different pathological types and different tumor sizes of lung cancers, a subgroup analysis of the model was performed in the whole group. First, IDH1 and one methylation DNA biomarker were selected to build two-marker models based on the whole cohort for further analysis. As shown in Fig. 2d, no significant differences were found between the primary three-marker panel and two other two-marker panels. However, the diagnostic abilities of the two-marker panels were significantly stronger than each enrolled individual biomarker (Additional file 1: Table S2, Additional file 2: Figures S1–2).

Second, a tumor size of 5 cm was chosen for dividing the patients into subgroups of early stage (T1–2 stage) and advanced stage (T3–4 stage) according to the 8th TNM staging system. In the group of patients with early stage lung cancers, the diagnostic panel demonstrated significantly better performance than the two DNA methylated biomarkers, with an AUC of 0.832 (95% *CI* 0.771–0.881) (*p *< 0.05, Table 4). Furthermore, the AUC (0.967, 95% *CI* 0.909 − 0.992) of the 3-biomarker panel was significantly higher than IDH1 and the PTGER4 gene in patients with tumors larger than 5 cm (*p *< 0.0001 and *p *= 0.0038, respectively, Table 4). Among the individual biomarkers, IDH1 had the significantly highest AUC value compared with the two other methylation biomarkers in the group of early stage lung cancers (Fig. 2e, Additional file 1: Table S3). However, in the T3–4 stage group, the AUC values of the two enrolled methylated biomarkers were higher than 0.92 (Fig. 2f), showing significantly better discriminative ability in contrast to IDH1 (AUC: 0.731, 95% *CI* 0.732–0.815, Table 4).

**Table 4:** Subgroup analysis of the 3-biomarker model

Subgroups	Variables	AUC	S.E.	95% CI	*p* value
≤ 5 cm	shox2	0.643	0.0397	0.571–0.710	< 0.0001
	ep4	0.619	0.0377	0.547–0.688	0.0007
	idh1	0.79	0.0335	0.726–0.845	0.0903*
	panel	0.832	0.0297	0.771–0.881	–
>5 cm	shox2	0.958	0.0218	0.898–0.988	0.6006*
	ep4	0.924	0.0396	0.853–0.968	0.0038
	idh1	0.731	0.0552	0.632–0.815	< 0.0001
	panel	0.967	0.016	0.909–0.992	–
Ade	shox2	0.629	0.0413	0.555–0.699	< 0.0001
	ep4	0.618	0.0389	0.544–0.689	< 0.0001
	idh1	0.791	0.0342	0.724–0.847	0.2415*
	panel	0.819	0.0315	0.756–0.872	–
non-Ade	shox2	0.907	0.0355	0.837–0.954	0.0563*
	ep4	0.838	0.0473	0.755–0.902	0.0034
	idh1	0.748	0.0467	0.656–0.826	< 0.0001
	panel	0.963	0.0161	0.909–0.990	–

*shox2* short stature homeobox 2 gene, *ep4* prostaglandin E2 receptor 4 gene (PTGER4), *idh1* isocitrate dehydrogenase 1, *AUC* area under the curve, *S.E.* standard error, *95% CI* 95% confidence interval

* Nonsignificant difference

Additionally, the diagnostic abilities of our panel in different pathological types of lung cancers were assessed. As the proportion of lung Ade was higher than other subtypes in the studied population, patients were divided into two groups of Ade and non-Ade lung cancers. As shown in Table 4, the panel showed significantly stronger diagnostic ability (AUC: 0.963, 95% *CI* 0.909–0.990) in diagnosing patients with non-Ade than the PTGER4 and IDH1 biomarkers, with *p *= 0.0034 and *p *< 0.0001, respectively (Fig. 2g). And, the AUC value of IDH1 (AUC: 0.791, 95% *CI* 0.724–0.847) was lower than that of the established three-biomarker combination (AUC: 0.819, 95% *CI* 0.756–0.872) in the lung Ade group, but nonsignificantly (*p *= 0.2415, Fig. 2h). The individual comparisons of single biomarkers demonstrated that the IDH1 biomarker had significantly lower efficacy in discriminating patients with SCC or SCLC than the DNA methylation of the SHOX2 gene (*p *= 0.0106, Additional file 1: Table S3).

## Discussion

Although the substantial improvements of radiological technology and advanced clinical treatment options, such as the shift from chest X-ray to high-resolution computed tomography (HRCT), have contributed to the progress of the curative modality of lung cancer, lung cancer patients are generally diagnosed at advanced stages [26] with a poor survival rate [4, 27]. Additionally, Ade has exceeded SCC, becoming the most common histological subtype of lung cancer [28, 29] due to the design of cigarette filter ventilation and changes in the composition of cigarettes [29–31]. Thus, understanding the epidemiological changes in lung cancers is essential for building a diagnostic tool with stability and comprehensive ability for discriminating lung cancers in the general population.

Based on our previous study, the diagnostic effectiveness of IDH1 in lung cancers, especially in patients with Ade, has been validated. However, its plasma level in SCC was significantly lower than that in Ade [25] and was uncertain when compared with that in SCLC. Since the result of the low predictive value of individual candidate biomarkers could be enhanced by combination [32–34], IDH1 was chosen to construct a panel to discriminate lung cancers as a candidate biomarker of the most common lung Ade. On the other hand, almost all cancers exhibit altered DNA methylation, an epigenetic marker that contributes to cancer development [35] and progression [36], making these epigenetic events a source of biomarkers and means for the detection of cancers [37, 38]. Therefore, the DNA methylation of the SHOX2 gene has been qualified as a sensitive biomarker for lung cancer diagnosis and staging, specifically for SCC and SCLC [14, 15, 17, 39], accompanied by an analysis of the methylation level of PTGER4 in our study. Moreover, the AUC value of IDH1 in both the training and validation groups was nonsignificantly higher than that of any other biomarkers, except for the levels of IDH1 and methylated PTGER4 DNA in the training group (AUCs of 0.781 and 0.67, respectively, *p *= 0.0351, Additional file 1: Table S4). Between the two methylation DNA biomarkers, nonsignificant differences were observed either in the training and validation cohorts (Additional file 1: Table S4). Although no significant differences were found between our primary 3-marker model and the other two 2-marker models (Fig. 2d), however in the subgroup analysis of non-Ade lung cancers, the primary 3-marker showed a significantly higher value of AUC than that of the other two 2-marker panels as shown in the Additional file 1: Table S5, Additional file 3: Figure S3. These results indicate that the diagnostic efficacy of the three biomarkers was basically comparable and demonstrate the necessity of their combination. In the statistical analysis, logistic regression was performed on the randomly assigned training group, and the final configuration enrolled the three tested blood biomarkers for diagnosing lung cancers, which was validated in an independent group.

Prof. Gunter had confirmed the discriminative performance of the SHOX2/PTGER4 DNA methylation marker panel with AUC values of 0.91–0.98, in a population with up to 61% non-Ade lung cancers [23]. In contrast, the ratio of Ade lung cancer and non-Ade lung cancers in our training group was nearly 4:1 (Ade:non-Ade, 90:25) indicating that our panel was more consistent with the current epidemiological changes of lung cancers [28, 29]. A further analysis of the single enrolled biomarkers, as shown in Fig. 2e–h, implied high specificity in groups of different tumor stages and histological types. Elevated expression levels of IDH1 in tissues of earlier-stage lung cancers have been previously confirmed, but no significant difference was found between Ade and SCC [24]. Therefore, further studies are needed to explore the reason for the high specificity of IDH1 in Ade lung cancers. On the other hand, the relatively stronger stage-specificity of methylation biomarkers in advanced-stage lung cancers may be explained by the higher amount of cell-free DNA in the bloodstream of patients with larger-sized tumors [14]. Additionally, as SCC and SCLC are commonly located centrally in the hilum, non-Ade lung cancers are more likely to invade large blood vessels [40–42]. As a consequence, higher levels of candidate DNA methylation will be detected by the blood test compared to the peripherally located Ade lung cancers in the lung lobes. With attributable risks of 85% and 60% to lung cancers in males and females caused by cigarette smoking [2, 43], respectively, the association between alterations of DNA methylation status and tobacco smoking has also been discussed and studied [44, 45]. Hence, we speculate that the strength of the predictive model may be increased when adding the smoking habit as a covariate in the final configuration.

Interestingly, the ROC curve of our primary 3-marker model almost overlapped with those of the other two 2-marker panels (combination of IDH1 and one single methylation biomarker), as shown in Fig. 2d. However, the 3-marker panels showed significantly stronger diagnostic performance compared with any 2-marker model in the analysis of non-Ade group (Additional file 1: Table S5, Additional file 3: Figure S3) demonstrating the advantage of a combined assessment over individual marker tests. In addition, based on the outstanding performance of our 3-marker model (AUC of 0.905) in the validation cohort, the probability of overfitting could be excluded as well. In our study, Youden’s index was used for the assessment of our diagnostic model. Although the specificity in the training group was below 70%, the PPVs in the training and validation groups were as high as 84.6% and 93.3%, respectively. Because radiological technology has been developed to provide more useful information, a comprehensive assessment of CT information and blood biomarkers may further enhance the PPV. In addition, healthy controls who were misclassified as the positive group should be closely followed-up with annual examinations in cases of lung cancers appearing in the absence of symptoms.

At the aspect of underlying molecular mechanisms of high diagnostic efficiency of wild-type IDH1 in NSCLC, we speculate that it is an important enzyme for redox state, DNA repair and epigenetic regulation in malignant tumors and its activation through mutation or overexpression plays a critical role in the initiation and development of different cancers, including NSCLC. Furthermore, we suggest that the combination of plasma IDH1 levels and DNA methylation levels of SHOX2 and/or PTGER4 showed better efficiency because the overexpression of wild-type IDH1 could produce alpha-ketoglutarate and its subsequent activation of DNA-demethylating enzymes such as TET2 might alter the DNA methylation levels of SHOX2/PTGER4 in NSCLC [46]. Therefore, the specific underlying mechanisms still need our further validation in the following researches.

## Conclusions

Individual biomarkers of good stage-specificity and histological type specificity were confirmed and combined in our study. Additionally, our newly established panel showed highly significantly stronger diagnostic efficacy compared with the enrolled single factors. Thus, the diagnostic efficacy of lung cancers could be improved by testing blood markers in conjunction.

## Limitations

Some limitations existed in our study as follows. First, a smoking history of healthy controls was not available, resulting in no case matching based on smoking habits. Second, our enrolled cohort was from a single medical institution, and a larger number of cohorts from multiple medical centers will be studied to eliminate underlying confounding variables in future studies. Third, blood levels of conventional biomarkers for lung cancers, such as CEA, CA125, CYFRA 21-1 or SCC, were not routinely tested. Consequently, the differences in the diagnostic value between our model and these commonly used markers could not be assessed.

## Supplementary information

**Additional file 1: Table S1.** Multivariable logistic regression. **Table S2.** Comparisons of the 2-marker models in the whole cohort. **Table S3.** Individual biomarker comparisons in subgroups of the whole cohort. **Table S4.** Comparisons between methylation DNA biomarkers in the training and validation cohorts. **Table S5.** Comparisons between the primary 3-marker model and the 2-maker models in the subgroup of non-Ade lung cancers.

**Additional file 2: Figure S1–S2.** ROCs of the 2-marker model in the whole cohort.

**Additional file 3: Figure S3.** ROCs of the primary 3-marker model and the 2-marker models in the non-Ade subgroup of the whole cohort.

## Data Availability

Not applicable.

## Abbreviations

Ade: adenocarcinoma
AUC: area under the curve
CA125: cancer antigen 125
CEA: carcinoembryonic antigen
CI: 95% confidence interval
CYFRA 21-1: cytokeratin 19 fragment
ep4: prostaglandin E2 receptor 4 gene (PTGER4)
HRCT: high-resolution computed tomography
IDH1: isocitrate dehydrogenase 1
LDCT: low-dose computed tomography
NLST: National Lung Screening Trial
NPV: negative predictive value
NSCLC: non-small cell lung cancer
PPV: positive predictive value
ROC: receiver operating characteristic
SCC: squamous cell carcinoma
SCLC: small cell lung cancer
Se: sensitivity
SHOX2: short stature homeobox 2 gene
Sp: specificity
SD: standard deviation
YI: Youden’s index
