# Supplementary material for: Translational value of IDH1 and DNA methylation biomarkers in diagnosing lung cancers: a novel diagnostic panel of stage and histology-specificity
Source: J Transl Med. 2019 Dec 30;17:430. doi: 10.1186/s12967-019-2117-7 (PMC6936123; doi:10.1186/s12967-019-2117-7)
Supplement: Supplementary file 1 — Additional file 1: Table S1. Multivariable logistic regression. Table S2. Comparisons of the 2-marker models in the whole cohort. Table S3. Individual biomarker comparisons in subgroups of the whole cohort. Table S4. Comparisons between methylation DNA biomarkers in the training and validation cohorts. Table S5. Comparisons between the primary 3-marker model and the 2-maker models in the subgroup of non-Ade lung cancers. [file 12967_2019_2117_MOESM1_ESM.docx]

| Variables | B | S.E. | Wald | F | Sig | Exp(B) |
| --- | --- | --- | --- | --- | --- | --- |
| shox2 | -0.147 | 0.068 | 4.716 | 1 | 0.03 | 0.863 |
| ep4 | -0.28 | 0.137 | 4.181 | 1 | 0.041 | 0.756 |
| idh1 | 0.435 | 0.098 | 19.814 | 1 | <0.001 | 1.544 |
| Constant | 16.821 | 5.899 | 8.131 | 1 | 0.004 | 20198714.8 |

Table S1 Multivariable logistic regression.

Abbreviations: shox2 = short stature homeobox 2 gene; ep4 = prostaglandin E2 receptor 4 gene (PTGER4); idh1 = isocitrate dehydrogenase 1; B = beta coefficient; S.E. = standard error; F = degrees of freedom; Sig = significance; Exp(B) = exponent of B.

Table S2 Comparisons of the 2-marker models in the whole cohort.

| Group | Variables | AUC | S.E. | 95% CI | *p* value |
| --- | --- | --- | --- | --- | --- |
|  | shox2 | 0.7 | 0.0351 | 0.634 - 0.759 | < 0.0001 |
|  | idh1 | 0.78 | 0.033 | 0.719 - 0.833 | 0.0095 |
| Whole group | shox2+idh1 | 0.834 | 0.029 | 0.779 - 0.881 | / |
| (n=221) | ep4 | 0.674 | 0.0338 | 0.608 - 0.735 | < 0.0001 |
|  | idh1 | 0.78 | 0.033 | 0.719 - 0.833 | 0.0009 |
|  | ep4+idh1 | 0.846 | 0.0277 | 0.792 - 0.891 | / |

Receiver operating characteristic (ROC) curves for each single tested biomarker and 2-marker models in whole cohort. Abbreviations: shox2 = short stature homeobox 2 gene; ep4 = prostaglandin E2 receptor 4 gene (PTGER4); idh1 = isocitrate dehydrogenase; AUC = area under the curve; S.E. = standard error; 95% *CI* = 95% confidence interval.

Table S3 Individual biomarker comparisons in subgroups of the whole cohort.

| Subgroups | Variables | AUC | S.E. | 95% *CI* | *p* value | |
| --- | --- | --- | --- | --- | --- | --- |
| ≤5 cm | shox2 | 0.643 | 0.0397 | 0.571-0.710 | / | 0.0038**^△^** |
|  | ep4 | 0.619 | 0.0377 | 0.547-0.688 | 0.6184 | 0.0007**^△^** |
|  | idh1 | 0.79 | 0.0335 | 0.726-0.845 | 0.0038**^△^** | / |
| >5 cm | shox2 | 0.958 | 0.0218 | 0.898-0.988 | / | 0.0001**^△^** |
|  | ep4 | 0.924 | 0.0396 | 0.853-0.968 | 0.3642 | 0.0038**^△^** |
|  | idh1 | 0.731 | 0.0552 | 0.632-0.815 | 0.0001**^△^** | / |
| Ade | shox2 | 0.629 | 0.0413 | 0.555-0.699 | / | 0.0021**^△^** |
|  | ep4 | 0.618 | 0.0389 | 0.544-0.689 | 0.8305 | 0.0007**^△^** |
|  | idh1 | 0.791 | 0.0342 | 0.724-0.847 | 0.0021**^△^** | / |
| non-Ade | shox2 | 0.907 | 0.0355 | 0.837-0.954 | / | 0.0106**^△^** |
|  | ep4 | 0.838 | 0.0473 | 0.755-0.902 | 0.057 | 0.2271 |
|  | idh1 | 0.748 | 0.0467 | 0.656-0.826 | 0.0106**^△^** | / |

Abbreviations: shox2 = short stature homeobox 2 gene; ep4 = prostaglandin E2 receptor 4 gene (PTGER4); idh1 = isocitrate dehydrogenase 1; Ade = adenocarcinoma; non-Ade = non-adenocarcinoma lung cancer; AUC = area under the curve; S.E. = standard error; 95% *CI* = 95% confidence interval; **^△^** = significant difference.

Table S4. Comparisons between methylation DNA biomarkers in the training and validation cohorts

| Subgroups | Variables | AUC | S.E. | 95% *CI* | *p* value | |
| --- | --- | --- | --- | --- | --- | --- |
| Training  Cohort | shox2 | 0.714 | 0.0398 | 0.640 - 0.781 | 0.1966 | / |
|  | ep4 | 0.67 | 0.0391 | 0.594 - 0.740 | 0.0351**^△^** | 0.3507 |
|  | idh1 | 0.781 | 0.0385 | 0.711 - 0.841 | / | 0.1966 |
| Validation  Cohort | shox2 | 0.659 | 0.0767 | 0.513 - 0.786 | 0.3855 | / |
|  | ep4 | 0.699 | 0.0684 | 0.555 - 0.820 | 0.6151 | 0.6165 |
|  | idh1 | 0.755 | 0.0703 | 0.615 - 0.865 | / | 0.3855 |

Abbreviations: shox2 = short stature homeobox 2 gene; ep4 = prostaglandin E2 receptor 4 gene (PTGER4); idh1 = isocitrate dehydrogenase 1; AUC = area under the curve; S.E. = standard error; 95% *CI* = 95% confidence interval; **^△^** = significant difference.

Table S5. Comparisons between the primary 3-marker model and the 2-maker models in the subgroup of non-Ade lung cancers

| Subgroup | Variables | AUC | S.E. | 95% *CI* | *p* value |
| --- | --- | --- | --- | --- | --- |
| non-Ade | 3-marker model | 0.963 | 0.0161 | 0.909 to 0.990 | / |
|  | ep4+idh1 model | 0.927 | 0.0237 | 0.861 to 0.968 | 0.0156 **^△^** |
|  | shox2+idh1mode | 0.929 | 0.0236 | 0.864 to 0.970 | 0.0093 **^△^** |

Abbreviations: shox2 = short stature homeobox 2 gene; ep4 = prostaglandin E2 receptor 4 gene (PTGER4); idh1 = isocitrate dehydrogenase 1; non-Ade = non-adenocarcinoma lung cancer; AUC = area under the curve; S.E. = standard error; 95% *CI* = 95% confidence interval; **^△^** = significant difference.
