# Supplementary material for: Translational value of IDH1 and DNA methylation biomarkers in diagnosing lung cancers: a novel diagnostic panel of stage and histology-specificity
Source: J Transl Med. 2019 Dec 30;17:430. doi: 10.1186/s12967-019-2117-7 (PMC6936123; doi:10.1186/s12967-019-2117-7)
Supplement: Supplementary file 3 — Additional file 3: Figure S3. ROCs of the primary 3-marker model and the 2-marker models in the non-Ade subgroup of the whole cohort. [file 12967_2019_2117_MOESM3_ESM.docx]

Figure S3. ROCs of the primary 3-marker model and the 2-marker models in the non-Ade subgroup of the whole cohort.
